# Molecular Characterization of the Fecal Microbiota in Patients with Nonalcoholic Steatohepatitis – A Longitudinal Study

**DOI:** 10.1371/journal.pone.0062885

**Published:** 2013-04-25

**Authors:** Vincent Wai-Sun Wong, Chi-Hang Tse, Tommy Tsan-Yuk Lam, Grace Lai-Hung Wong, Angel Mei-Ling Chim, Winnie Chiu-Wing Chu, David Ka-Wai Yeung, Patrick Tik-Wan Law, Hoi-Shan Kwan, Jun Yu, Joseph Jao-Yiu Sung, Henry Lik-Yuen Chan

**Affiliations:** 1 Department of Medicine and Therapeutics, The Chinese University of Hong Kong, Hong Kong; 2 Institute of Digestive Disease, The Chinese University of Hong Kong, Hong Kong; 3 Department of Zoology, University of Oxford, Oxford, United Kingdom; 4 Department of Imaging and Interventional Radiology, The Chinese University of Hong Kong, Hong Kong; 5 Department of Clinical Oncology, The Chinese University of Hong Kong, Hong Kong; 6 School of Life Sciences, The Chinese University of Hong Kong, Hong Kong; University of Tor Vergata, Italy

## Abstract

**Background:**

The human gut microbiota has profound influence on host metabolism and immunity. This study characterized the fecal microbiota in patients with nonalcoholic steatohepatitis (NASH). The relationship between microbiota changes and changes in hepatic steatosis was also studied.

**Methods:**

Fecal microbiota of histology-proven NASH patients and healthy controls was analyzed by 16S ribosomal RNA pyrosequencing. NASH patients were from a previously reported randomized trial on probiotic treatment. Proton-magnetic resonance spectroscopy was performed to monitor changes in intrahepatic triglyceride content (IHTG).

**Results:**

A total of 420,344 16S sequences with acceptable quality were obtained from 16 NASH patients and 22 controls. NASH patients had lower fecal abundance of *Faecalibacterium* and *Anaerosporobacter* but higher abundance of *Parabacteroides* and *Allisonella*. Partial least-square discriminant analysis yielded a model of 10 genera that discriminated NASH patients from controls. At month 6, 6 of 7 patients in the probiotic group and 4 of 9 patients in the usual care group had improvement in IHTG (P = 0.15). Improvement in IHTG was associated with a reduction in the abundance of Firmicutes (R^2^ = 0.4820, P = 0.0028) and increase in Bacteroidetes (R^2^ = 0.4366, P = 0.0053). This was accompanied by corresponding changes at the class, order and genus levels. In contrast, bacterial biodiversity did not differ between NASH patients and controls, and did not change with probiotic treatment.

**Conclusions:**

NASH patients have fecal dysbiosis, and changes in microbiota correlate with improvement in hepatic steatosis. Further studies are required to investigate the mechanism underlying the interaction between gut microbes and the liver.

## Introduction

Nonalcoholic fatty liver disease (NAFLD) is the most common chronic liver disease worldwide [1], [2]. Nonalcoholic steatohepatitis (NASH) is the progressive form of NAFLD and can result in cirrhosis and hepatocellular carcinoma [3]–[5]. Because of the close relationship between NASH and metabolic syndrome, NASH is also strongly associated with cardiovascular disease [6], [7].

While hepatic steatosis is the result of energy excess and abnormal lipid metabolism, the mechanism leading to NASH and liver injury is not completely understood. Several lines of evidence, however, suggest that the ‘gut-liver axis’ may contribute significantly to the pathogenesis of NASH. NAFLD/NASH patients have small intestinal bacterial overgrowth and increased intestinal permeability [8]. They also have increased blood level of bacterial endotoxin [9], [10]. Endotoxin, also known as lipopolysaccharide, is a major constituent of the outer cell membrane of Gram-negative bacteria. Endotoxin and free fatty acids are known to induce hepatic necroinflammation through its action on Toll-like receptors [11], [12]. Animal and small human studies show that probiotics and prebiotics may ameliorate NASH [13]–[17].

The human gut is home to around 100 trillion commensal organisms. The gut microbiome contains 10 times more genes than the human host [18]. Current knowledge on the association between gut microbiota composition and NASH is limited. Altered gut microbiota is associated with obesity and type 2 diabetes in animals and humans [19]–[22]. Transplantation of gut microbiota from normal mice to germ-free mice leads to substantial increase in body weight and hepatic steatosis [23]. Furthermore, healthy women receiving choline-deficient diet to induce fatty liver were found to have altered fecal composition of *Gammaproteobacteria* and *Erysipelotrichi* [24]. However, choline-deficient diet is a highly artificial intervention and cannot reflect the situation of NASH patients in the clinic. The gut microbiota composition of adult NASH patients has not been systematically studied.

In this study, we determined fecal microbiota changes in NASH patients. We also longitudinally studied the association between changes in fecal microbiota and changes in hepatic steatosis with time.

## Materials and Methods

### Study Design

This was a preplanned analysis of a randomized controlled trial in NASH patients. Details of the original trial have been reported previously [17]. In brief, 20 NASH patients were randomized to receive probiotic treatment (n = 10) or usual care (n = 10) for 6 months. Patients in the probiotic group received 1 sachet of Lepicol probiotic and prebiotic formula (Healthy Bowels Company Ltd, Birmingham, UK) twice a day. Each 10 g sachet contained 200 million probiotic cultures of *Lactobacillus plantarum* (ATCC 14917), *Lactobacillus delbrueckii ssp bulgaricus* (ATCC 11842), *Lactobacillus acidophilus* (ATCC 4356), *Lactobacillus rhamnosus* (ATCC 7469) and *Bifidobacterium bifidum* (ATCC 29521). Patients in the usual care group did not receive probiotic. Both groups were instructed to exercise for at least 90 minutes per week and consume low carbohydrate, low fat diet at baseline. At baseline and month 6, all patients underwent proton-magnetic resonance spectroscopy (^1^H-MRS) to measure intrahepatic triglyceride content (IHTG) and provided fresh fecal samples. This study focused on the fecal microbiota analysis of these NASH patients. This was further compared with the fecal microbiota of healthy volunteers without NAFLD.

### Ethics

The study was approved by the Ethics Committee of The Chinese University of Hong Kong (CRE-2008.258) and was registered at ClinicalTrials.gov (NCT00870012).

### Patients

Cases were patients aged 18 to 70 years with histology-proven NASH, defined as hepatic steatosis of more than 5% and inflammation with hepatocyte ballooning [25]. Controls were healthy volunteers with normal liver function tests and no history of liver diseases. Subjects in both groups had negative hepatitis B surface antigen, negative anti-hepatitis C virus antibody and anti-nuclear antibody titer below 1/160. Subjects with significant alcohol consumption (over 20 g per day in men or 10 g per day in women), liver decompensation or malignancy, or secondary causes of fatty liver (e.g. use of systemic steroids or methotrexate) were also excluded. All subjects provided written informed consent.

### Clinical Assessments

At baseline, anthropometric parameters including body weight, body height and waist circumference were measured in NASH patients and controls. Waist circumference was measured at a level midway between the lower rib margin and iliac crest with the tape all around the body in the horizontal position. Body mass index (BMI) was calculated as body weight (kg) divided by body height (m) squared. Blood was taken for liver biochemistry, glucose and lipids after fasting for at least 8 hours. Liver biopsy was scored according to the NASH Clinical Research Network system [26].

IHTG was measured by ^1^H-MRS as described previously [27]. The whole body 3.0 Tesla scanner (Philips Healthcare, Best, the Netherlands) with an echo time of 40 ms and repetition time of 5000 ms was used. All control subjects had IHTG below 5%. All the assessments were repeated at 6 months in the NASH group.

### Stool Sample Preparation

Early morning stool samples from subjects and controls were collected and stored at −80°C. 180 mg material was removed from the surface and core of the frozen stool samples, mixed manually, and the total DNA was purified by QIAamp DNA Stool Mini Kit (Qiagen, Inc, Hilden, Germany). All microbial community DNA samples were analyzed for 16S ribosomal RNA sequences by pyrosequencing. For each sample, 2 µl DNA was amplified by polymerase chain reaction (PCR) using forward primer (986F 5′WACGCGARGAACCTTACC3′) and reverse primer (1390R 5′TGACGGGCGGTGWGTAC3′), which annealed to V1–V2 variable regions of bacterial 16S ribosomal RNA gene [28], [29]. The PCR products were separated by 2% agarose gel electrophoresis, purified with the QIAquick PCR purification Kit (Qiagen) before pyrosequencing using GS FLX system (Genome Sequencer FLX system, 454 Life Sciences, Inc, Bradford, CT, USA).

Tag and primer sequences were removed from the amplicon reads. The sequences were then subject to quality screening, in which those with length between 400 bp and 475 bp (constituting >97.7% of reads), and with fewer than two ambiguity bases were kept. These sequences were clustered into operational taxonomic units (OTUs) by >97% similarity using UCLUST method in USEARCH v4.01. The most abundant sequence from each OTU was selected as a representative sequence and taxonomically classified by matching against the annotated bacterial and archeal 16S rRNA sequence databases (N = 1,921,179) in RDP Release 10, Update 27 [30], using Ribosomal Database Project naïve Bayesian Classifier with a 80% confidence threshold [31]. The sequences are available at http://www.ncbi.nlm.nih.gov/sra?term=SRA049741.

### Statistical Analysis

Continuous variables were expressed in mean ± standard deviation or median (interquartile range) and compared between the NASH and control groups using unpaired *t* test or Mann-Whitney’s test as appropriate. Changes in continuous variables from baseline to month 6 were assessed using paired *t* test. Categorical variables were compared using χ^2^ test or Fisher exact test as appropriate. Statistical significance was taken as a two-sided P value of less than 0.05.

Community richness and diversity in each sample was studied by biodiversity indices including Shannon’s index, inverse Simpson’s diversity, Pielou’s evenness, Chao-1 estimator computed from OTUs. These indices were estimated from a randomly rarefied dataset of 4,000 reads in each subject, because of their dependence on the size of sequence set. Bray-Curtis dissimilarities between samples were calculated and compared between different subject groups. Biodiversity measures were done using *vegan* package on R statistical program v2.11.1 (http://www.r-project.org/).

Relative microbial abundance in the NASH and control groups were compared at the phylum, class, order, family, genus, and OTU levels. Unpaired and paired *t* tests were used to justify the significant difference in biodiversity and the abundance of a microbial lineage between control and patients and in patients before and six months after intake of study medications, respectively. Correlations between the changes of IHTG content and abundances of bacterial groups were justified by Pearson’s correlation tests using the R program.

Partial least-square discriminant analysis (PLS-DA), implemented in MetaboAnalyst, was performed to discriminate the microbial community profiles between NASH and control subjects [32]. Autoscaling was applied. The NASH and healthy phenotypes represented two classes and OTU abundances were the predicting variables. Rare variables which were observed in <3 subjects and <5 reads in total were excluded from the analysis.

The OTU representative sequences were aligned using Infernal v1.0.2 [33], which were then subject to maximum likelihood phylogenetic analysis using FastTree v2.1 [34], with a general time reversible nucleotide substitution model. From the maximum likelihood phylogeny, the weighted and normalized UniFrac distance between each pair of samples was estimated in Fast UniFrac [35]. Principal component analysis was performed on the UniFrac distance metric.

## Results

Twenty NASH patients and 22 controls were enrolled from March to September 2009. After excluding 4 NASH patients with suboptimal sequencing quality, 16 NASH patients (7 from the probiotic group and 9 from the usual care group) and 22 controls were included in the final analysis. As expected, NASH patients had higher BMI and waist circumference, and were more likely to have diabetes and hypertension (Table 1). The age range was 37–69 years in the NASH group and 24–61 years in the control group.

**Table 1:** Clinical characteristics of subjects with and without NASH.

	NASH			P (Usual care vs probiotics)		Control		P (All NASH vs controls)		
Baseline	All	Usual care	Probiotics							
N	16	9	7			22				
Age (years)	51±9	56±9	46±6	0.030		44±10		0.016		
Male:Female	9∶7	4∶5	5∶2	0.36		9∶13		0.35		
BMI (kg/m^2^)	29.1±5.6	28.6±6.1	29.8±5.3	0.69		22.2±2.7		<0.001		
Waist circumference (cm)	98±13	98±15	100±10	0.76		79±9		<0.001		
ALT (IU/l)	80 (44, 94)	65 (42, 94)	81 (52, 99)	1.0		22 (17, 30)		<0.001		
AST (IU/l)	40 (26, 50)	33 (22, 44)	48 (31, 52)	0.32		20 (17, 24)		<0.001		
Fasting glucose (mmol/l)	6.2±1.1	6.4±1.0	5.9±1.3	0.46		5.1±0.5		0.001		
Total cholesterol (mmol/l)	4.8 (4.2, 5.4)	5.0 (4.0, 5.5)	4.7 (4.4, 5.2)	1.0		5.1 (4.6, 5.7)		0.74		
HDL-cholesterol (mmol/l)	1.3 (1.0, 1.4)	1.3 (1.0, 1.7)	1.2 (1.0, 1.4)	1.0		1.6 (1.5, 1.8)		0.002		
LDL-cholesterol (mmol/l)	2.6 (2.2, 3.2)	2.7 (2.0, 3.2)	2.4 (2.1, 3.3)	1.0		3.0 (2.6, 3.5)		0.48		
Triglycerides (mmol/l)	1.8 (1.3, 2.7)	1.7 (1.3, 2.2)	2.5 (1.5, 2.9)	1.0		1.1 (0.8, 1.3)		0.003		
Diabetes, n (%)	6 (38)	4 (50)	2 (29)		0.61		0		0.003	
Hypertension, n (%)	10 (63)	6 (67)	4 (57)		1.0		1 (5)		<0.001	
Sulfonylurea, n (%)	3 (19)	1 (11)	2 (29)		0.55		0		0.066	
Metformin, n (%)	6 (38)	4 (44)	2 (29)		0.63		0		0.003	
Thiazolidinedione, n (%)	0	0	0		1.0		0		1.0	
Insulin, n (%)	0	0	0		1.0		0		1.0	
Steatosis grade, 1/2/3	1/9/6	1/5/3	0/4/3	0.65		–		–		
Lobular inflammation, 0/1/2/3	4/11/1/0	2/7/0	2/4/1	0.45		–		–		
Ballooning, 0/1/2	0/15/1	0/8/1	0/7/0	1.0		–		–		
Fibrosis stage, 0/1/2/3/4	6/6/1/1/2	3/4/0/0/2	3/2/1/1/0	0.34		–		–		
IHTG by ^1^H-MRS (%)	17.5±5.8	16.8±6.4	18.5±5.1	0.56		1.6±1.2		<0.001		
**Month 6**										
BMI (kg/m^2^)	28.7±5.5	28.0±5.9	29.5±5.1	0.59		–		–		
Waist circumference (cm)	99±12	98±14	100±10	0.72		–		–		
ALT (IU/l)	60 (42, 77)	66 (46, 74)	53 (41, 81)	1.0		–		–		
AST (IU/l)	34 (28, 36)	35 (28, 39)	31 (27, 34)	0.32		–		–		
Fasting glucose (mmol/l)	6.2±0.9	6.5±0.7	5.7±1.0	0.080		–		–		
Total cholesterol (mmol/l)	4.7 (4.3, 5.6)	4.9 (4.2, 5.8)	4.6 (4.4, 5.1)	1.0		–		–		
HDL-cholesterol (mmol/l)	1.3 (1.0, 1.7)	1.4 (1.0, 1.8)	1.2 (1.0, 1.3)	0.32		–		–		
LDL-cholesterol (mmol/l)	2.6 (2.2, 2.9)	2.7 (2.0, 3.7)	2.6 (2.3, 2.7)	0.36		–		–		
Triglycerides (mmol/l)	1.6 (1.1, 2.3)	1.5 (1.1, 2.0)	1.6 (1.4, 3.2)	0.32		–		–		
IHTG by ^1^H-MRS (%)	15.1±5.9	15.6±6.9	14.6±4.8	0.75		–		–		

Continuous variables were expressed as mean ± standard deviation or median (interquartile range).

ALT, alanine aminotransferase; BMI, body mass index; HDL, high density lipoprotein; IHTG, intrahepatic triglyceride content; LDL, low density lipoprotein; NAFLD, nonalcoholic fatty liver disease; NASH, nonalcoholic steatohepatitis.

### Fecal Microbiota in NASH and Control Subjects

**Figure 1:**
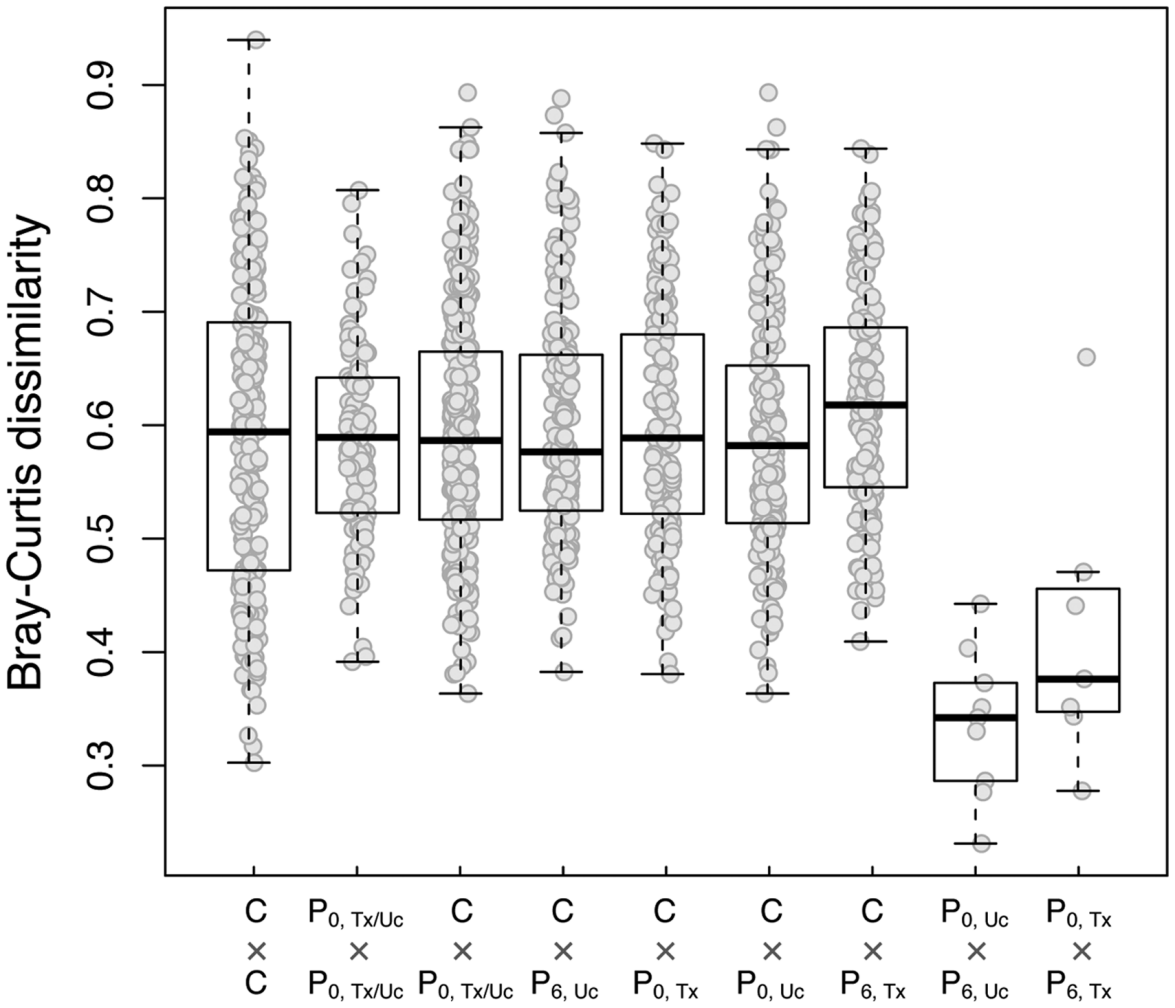
Genus level Bray-Curtis dissimilarity between fecal samples. Footnote: C, controls; P_0_, baseline samples of NASH patients; P_6_, month 6 samples of NASH patients; Uc, usual care group; Tx, probiotic treatment group.

A total of 420,344 16S sequences with acceptable quality were obtained, in which sufficient amount of DNA could be recovered. The average reads per sample was 7,784 (range 4,428–11,206). Based on sequence similarity of at least 97%, the total number of OTUs was 41,843, with an average of 775 OTUs per sample (range 285–1,280). The mean number of OTUs was 792 (512–1,075) in NASH patients and 810 (315–1,280) in controls (P = 0.80). The biodiversity indices and community distance summarized from the microbiota of the two groups were not significantly different (Table 2, Figure 1).

**Table 2:** Bacterial biodiversity in NASH patients and controls.

		NASH (Baseline)			NASH (Month 6)	
	Control	Total	Usual care	Probiotics	Usual care	Probiotics
N	22	16	9	7	9	7
Number of reads	8102±1407	7937±2036	7779±2300	8140±1796	7309±1488	7048±818
Number of OTUs	810±255	792±182	860±175	704±162	772±207	631±250
Shannon’s index	4.75±0.77	4.77±0.59	4.95±0.58	4.53±0.56	4.72±0.6	4.55±0.65
Inverse Simpson’s diversity index	33.19±23.1	34.16±22.37	41.67±25.47	24.52±13.88	33.24±19.13	31.05±19.25
Pielou’s evenness index	0.71±0.08	0.72±0.07	0.73±0.07	0.69±0.07	0.71±0.07	0.71±0.06
Chao-1 estimators	2231±865	2105±630	2357±565	1781±592	2180±649	1540±680

**Figure 2:**
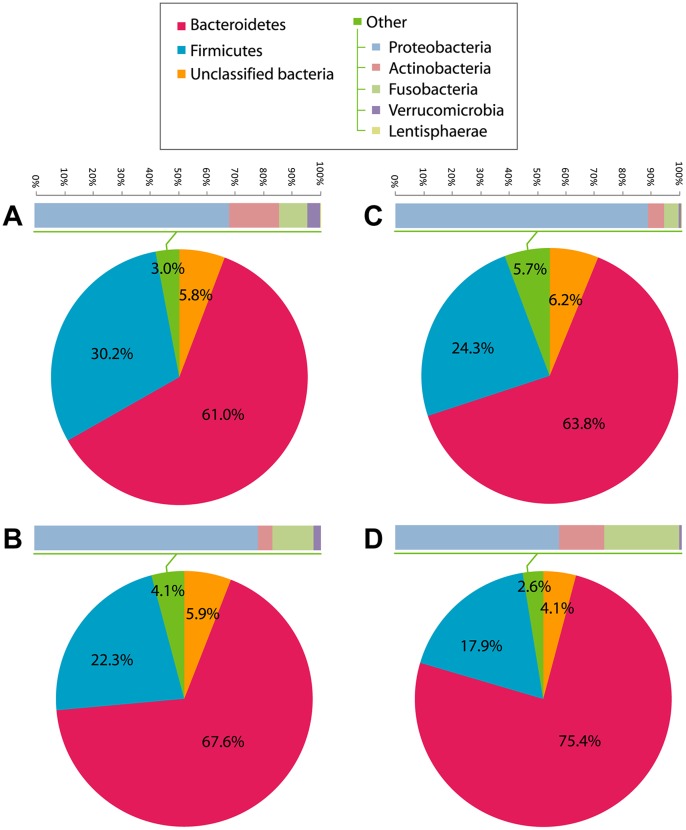
Relative abundance of bacterial phyla. (A) Controls (N = 22), (B) NASH patients at baseline (N = 16), (C) NASH patients at month 6 of usual care (N = 9), and (D) NASH patients at month 6 of probiotic treatment (N = 7).

**Figure 3:**
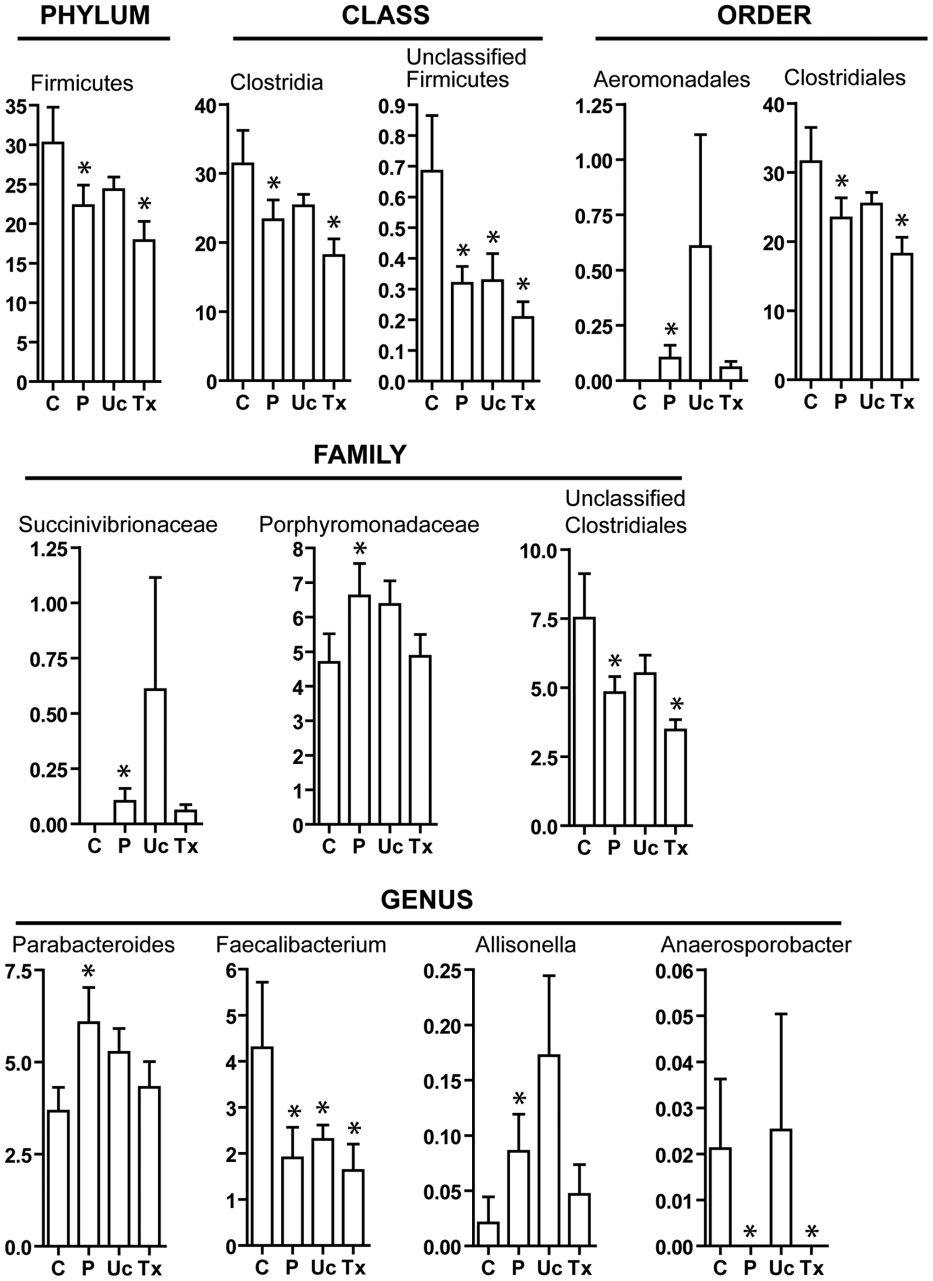
Abundance of bacterial clades that differed between controls and NASH patients. Footnote: Relative abundance is shown in percentage. C, controls; P, NASH patients; Uc, usual care group at month 6; Tx, probiotic treatment group at month 6. *<0.05 compared to controls.

In both NASH patients and controls, Bacteroidetes (67.6% vs 61.0%, P = 0.13) was the most abundant phylum in the fecal microbiota (Figure 2). The second most abundant phylum Firmicutes was significantly more enriched in controls than NASH patients (30.3% vs 22.3%, P = 0.029) (Figure 3). The order Aeromonadales, the families Succinivibrionaceae and Porphyromonadaceae, and the genera *Parabacteroides* and *Allisonella* were more abundant in NASH patients than controls (Figure 3). On the other hand, the class Clostridia, the order Clostridiales, and the genera *Faecalibacterium* and *Anaerosporobacter* were less abundant in NASH patients. Unclassified Firmicutes and Clostridiales were also less abundant in NASH patients.

**Figure 4:**
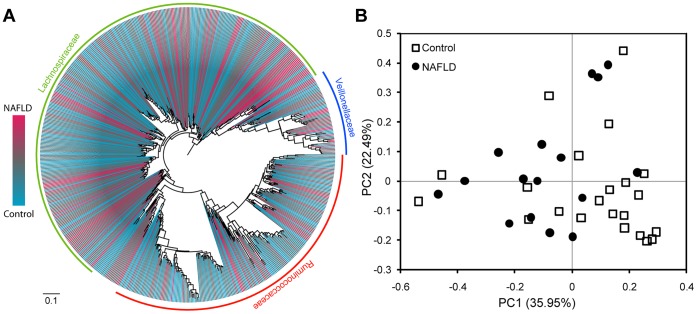
Firmicutes phylogeny and principal component analysis (PCA) plot based on Unifrac distances between the Firmicutes sequences in control and NASH subjects. (A) The Firmicutes phylogeny was reconstructed from the OTU representative sequences in the control and NASH samples, and their relative abundance was indicated by gradient color from red to blue. (B) PCA plot of controls and NASH patients. The percentage of variation explained by each principal component was indicated in the parenthesis.

Principal component analysis based on the Unifrac distances, which is a measure of phylogenetic relatedness between samples of microbial taxa, demonstrated no clear distinction in the whole microbial 16S sequence sets between controls and NASH (data not shown), yet showed some degree of phylogenetic separation between the Firmicutes sequences in controls and NASH patients (Figure 4), which was in accord with the analysis of relative abundance above.

**Figure 5:**
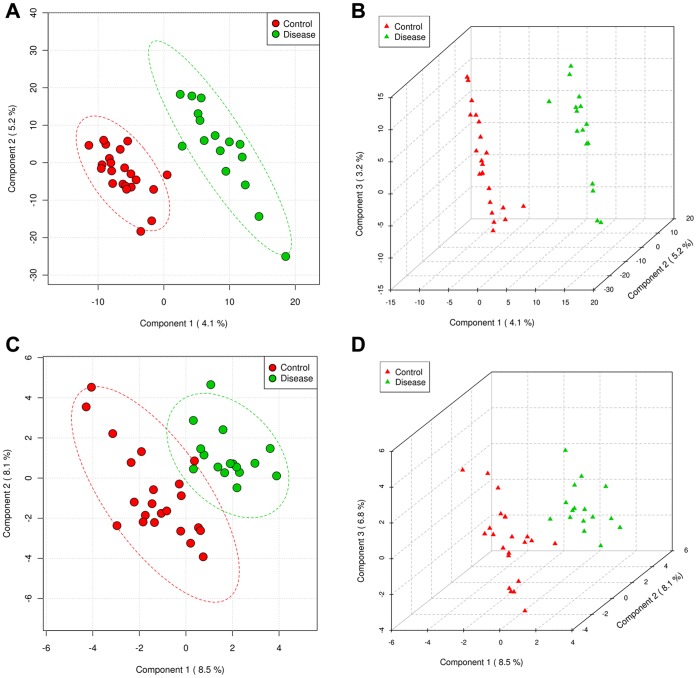
Score plots of PLS-DA distinguishing between the microbial community data of controls and NASH patients. (A and B) OTU level, (C and D) genus level.

The multivariate analysis method, PLS-DA, was used to identify the key bacterial clades responsible for the differentiation between control and NASH subjects. At the level of OTU abundance, score plots based on the 2^nd^ and 3^rd^ components showed that the two groups were well separated (Figure 5). The R^2^Y of this model at the first 3 components (with the best Q^2^) was 0.94 and 0.98, indicative of very good performance for the differentiation. However, the low Q^2^ value (<0.1) of this model suggested a poor performance of predicting the disease, which might be possibly due to relatively small sample size of NASH patients. By fitting the model to the abundance at the genus level, Q^2^ value was increased to 0.43 at the first 3 components. In this model, the ten bacterial genera with the highest variable importance in projection (VIP; VIP = 2.0–1.1), which reflect the contribution to discrimination, were *Parabacteroides*, *Faecalibacterium*, *Anaerofilum*, unclassified *Succinivibrionaceae*, unclassified *Porphyromonadaceae*, *Allisonella*, *Blautia*, *Anaerosporobacter*, *Lachnobacterium* and unclassified *Erysipelotrichaceae*. *Parabacteroides*, *Faecalibacterium*, *Allisonella* and *Anaerosporobacter* were also congruently identified by t-tests (Figure 3).

### Fecal Microbiota and Changes in Hepatic Steatosis at Month 6

All 16 NASH patients with good quality fecal microbiota sequences had repeated fecal analysis at 6 months. IHTG decreased by 4.0±5.5% in the probiotic group and 1.2±5.1% in the usual care group by month 6 (P = 0.31). Six of 7 patients in the probiotic group and 4 of 9 patients in the usual care group had improvement in IHTG (P = 0.15). Details of the other treatment outcomes have been reported previously [17]. As expected, paired samples from the same groups had much less dissimilarity from each other than samples from different groups (Figure 1). There was no significant change in bacterial biodiversity over time in both the probiotic group and the usual care group (Table 2).

**Figure 6:**
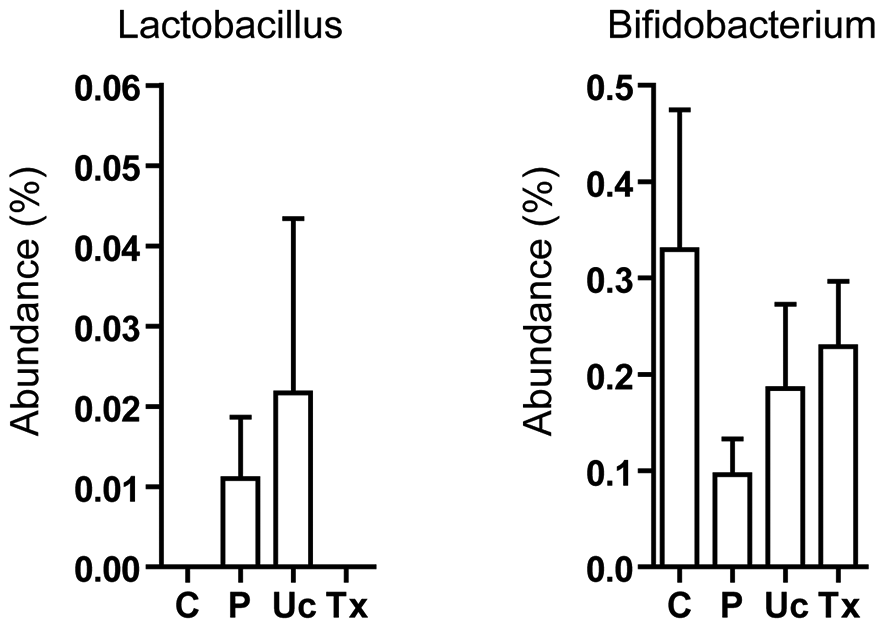
Abundance of *Lactobacillus* and *Bifidobacterium*. *Lactobacillus* and *Bifidobacterium* were the two bacterial genera contained in the probiotics used in this study. ‘C’, ‘P’, ‘Uc’ and ‘Tx’ refer to controls, NASH patients at baseline, NASH patient at 6 months after usual care and treatment of probiotic, respectively. There is no significant difference between each pair of study groups.

In the probiotic group, changes in bacterial abundance towards that of healthy controls were observed in the family Porphyromonodaceae (from 6.6% to 4.9%; vs 4.7% in controls; P = 0.84), its genus member *Parabacteroides* (from 6.1% to 4.3%; vs 3.7% in controls; P = 0.48), and another genus *Allisonella* (belonging to the Veillonellaceae family; from 0.09% to 0.05%; vs 0.02% in controls; P = 0.46) (Figure 3). Nevertheless, there was no significant increase in the abundance of *Lactobacillus* and *Bifidobacterium* after probiotic treatment (Figure 6).

Moreover, there was increased abundance of the order Aeromonadales and the family Succinivibrionaceae in the usual care group at month 6 (Figure 3). However, this was due to exceptionally high abundance of Succinivibrionaceae (and hence Aeromonadales; 4.6%) in 1 patient in the usual care group, which was very different from the other patients (0–0.7%). Second, the increased abundance of the genus *Anaerosporobacter* in the usual care group at month 6 was due to its presence in 1 patient but absence in all other NASH patients. The phenomenon was only observed in 1 outlier and involved only low abundance bacteria, and should therefore have limited pathological significance.

**Figure 7:**
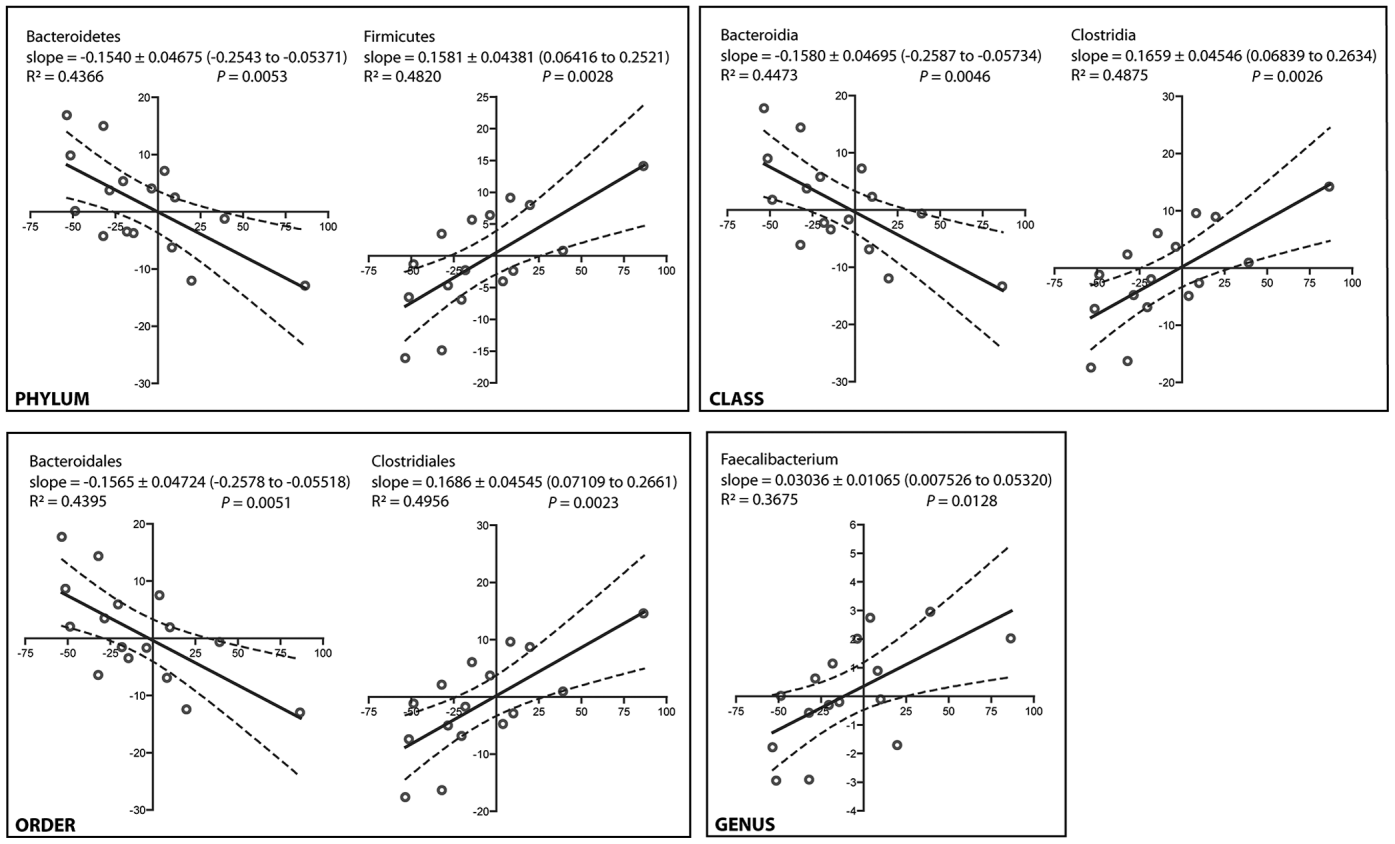
Correlation between the changes in intrahepatic triglyceride content and fecal bacterial abundance in 16 NASH patients over 6 months. Footnote: The x-axis and y-axis represents changes of intrahepatic triglyceride content and abundance of the indicated bacterial groups in 6 months, respectively. Solid and dashed lines are linear regression fits and 95% confidence bands, respectively.

In addition, fecal microbiota changes in the 16 NASH patients correlated with changes in IHTG at month 6. At the phylum level, a reduction in IHTG was accompanied by a reduction in the abundance of Firmicutes and an increase in Bacteroidetes (Figure 7). Improvement in hepatic steatosis was also associated with reduced abundance of the class Clostridia, the order Clostridiales and the genus *Faecalibacterium*. In contrast, reduced IHTG level was associated with increased abundance of the class Bacteroidia and the order Bacteroidales.

## Discussion

Cumulating data suggest that the human gut microbiota has profound influence on host metabolism and immunity. In this study, NASH patients demonstrated fecal dysbiosis but not significant changes in biodiversity. In addition, changes in fecal microbiota over time correlate with changes in hepatic steatosis.

With the recent development in molecular techniques, evaluation of thousands of DNA sequences can be done rapidly and accurately. The pyrosequencing method adopted in the current study has been shown to be as reliable as cloning [36]. Gut microbial dysbiosis is associated with various disorders such as inflammatory bowel disease and irritable bowel syndrome [37], [38]. Changes in gut microbiota have also been observed in obese animals with NASH [39], [40]. In our study, it is possible to distinguish NASH patients and healthy people at the OTU level based on a combination of 10 genera. Moreover, through a longitudinal study design, we found that changes in the fecal microbiota composition correlate well with the changes in hepatic steatosis in 6 months. For instance, obese patients were found to have higher abundance of Firmicutes and lower abundance of Bacteroidetes in previous studies [19]. Echoing previous findings, we found that NASH patients with improvement in hepatic steatosis actually had reduced abundance of Firmicutes and increased abundance of Bacteroidetes with time. On the other hand, the abundance of Firmicutes was unexpectedly lower in NASH patients than controls. It is unclear if the different pattern may be due to different dietary compositions in Chinese subjects.

In a recent study, children with biopsy-proven NASH had significant difference in gut microbiota from those with obesity alone [41]. Both groups were in turn different from non-obese controls. This suggests that gut microbiota may have specific influence on the NASH phenotype. That said, it should be highlighted that like all human association studies, causal relationship cannot be firmly established despite a trend toward dose-dependent relationship. Future animal studies are required to establish the causal relationship and work out the mechanism of the association.

At the genus level, *Parabacteroides*, *Faecalibacterium*, *Allisonella* and *Anaerosporobacter* were consistently found to be different between NASH patients and controls by *t* test and PLS-DA. *Faecalibacterium* was reduced by more than half in NASH subjects. *F. prausnitzii* is an anti-inflammatory commensal and is reduced in both patients with inflammatory bowel disease and irritable bowel syndrome [37], [38], [42]. Peripheral blood mononuclear cells have reduced expression of interleukin-12 and interferon-gamma and increased secretion of interleukin-10 when exposed to *F. prausnitzii* [42]. The significance of the other microbes identified in this study warrants further investigation.

Although probiotics have been shown to be beneficial in a number of animal and human NASH studies, their mechanism of action remains poorly understood [13]–[17]. In mouse studies, probiotics reduce the expression of proinflammatory cytokines and suppress natural killer T cells in the liver [13], [14], [43]. Since NASH is associated with endotoxemia, this may be another treatment target by probiotics [11], [12]. Based on our data, however, modification of colonic microbiota is unlikely to be the mechanism underlying the effect of probiotics. Ideally, biopsy samples along different parts of the gastrointestinal tract may provide deeper insights and are preferable to fecal samples. However, this would require repeated small bowel enteroscopy and cannot be justified in otherwise healthy individuals.

Our study has a few limitations. First, the sample size was relatively small. However, we improved the breadth and depth of data by evaluating an average of almost 8,000 reads of 16S sequences per sample. Our study is unique in having serial assessment of hepatic steatosis and fecal microbiota. All NASH patients also had liver biopsy at baseline. Furthermore, the changes in major bacterial phyla observed in this study were in accordance to the current understanding of the effect of those microbes. Second, all subjects were ethnic Chinese and the findings cannot be directly extrapolated to other populations. However, substantial inter-individual variation in gut microbiota exists even among people within the same nation [44]. While further confirmatory studies from other countries are welcomed, our study demonstrated the close relationship between gut microbiota and NASH.

In conclusion, NASH patients have gut microbial dysbiosis, and changes in microbiota correlate with improvement in hepatic steatosis. Further studies are required to investigate the mechanism underlying the interaction between gut microbes and the liver.
